# Introduction of Inactivated Poliovirus Vaccine and Trivalent Oral Polio Vaccine/Bivalent Oral Polio Vaccine Switch in the African Region

**DOI:** 10.1093/infdis/jiw616

**Published:** 2017-07-01

**Authors:** Carol Tevi-Benissan, Joseph Okeibunor, Gaël Maufras du Châtellier, Afework Assefa, Joseph Nsiari-Muzenyi Biey, Dah Cheikh, Messeret Eshetu, Blanche-Philomene Anya, Halima Dao, Yusuf Nasir, Bartholomew Dicky Akanmori, Richard Mihigo

**Affiliations:** 1 Immunization and Vaccines Development Programme, Family and Reproductive Health Cluster, and; 2 Polio Eradication Programme, Office of the Regional Director, World Health Organization (WHO) Regional Office for Africa, Brazzaville, Congo;; 3 United Nations Children’s Fund (UNICEF) Regional Office for West and Central Africa, Dakar, Senegal;; 4 UNICEF Regional Office for East and Southern Africa, Nairobi, Kenya;; 5 Immunization and Vaccines Development Programme, WHO Intercountry Support Team for West Africa, Ouagadougou, Burkina Faso;; 6 Immunization and Vaccines Development Programme, WHO Intercountry Support Team for Central Africa, Libreville, Gabon; and; 7 Immunization and Vaccines Development Programme, WHO Intercountry Support Team for East and Southern Africa, Harare, Zimbabwe

**Keywords:** switch, tOPV/bOPV, IPV, strengthened routine immunization, African region.

## Abstract

The Polio Eradication and Endgame Strategic plan outlines the phased removal of oral polio vaccines (OPVs), starting with type 2 poliovirus–containing vaccine and introduction of inactivated polio vaccine in routine immunization to mitigate against risk of vaccine-associated paralytic polio and circulating vaccine-derived poliovirus. The objective includes strengthening routine immunization as the primary pillar to sustaining high population immunity. After 2 years without reporting any wild poliovirus (July 2014–2016), the region undertook the synchronized switch from trivalent OPV (tOPV) to bivalent OPV (bOPV) as recommended by the Strategic Advisory Group of Experts on Immunization. Consequently the 47 countries of the World Health Organization (WHO) African Region switched from the use of tOPV to bOPV within the stipulated period of April 2016. Planning started early, routine immunization was strengthened, and technical and financial support was provided for vaccine registration, procurement, destruction, logistics, and management across countries by WHO in collaboration with the United Nations Children’s Fund (UNICEF) and partners. National commitment and ownership, as well as strong coordination and collaboration between UNICEF and WHO and with partners, ensured success of this major, historic public health undertaking.

Since the initiation of the eradication of poliomyelitis, the World Health Organization (WHO) has been making preparations in the African region for changes anticipated in immunization. Consequently, in 2006, the Working Group on Pre- and Post-Polio Eradication Issues was established to examine all the issues related to the pre– and post–polio eradication eras [1]. In its final report the expert committee recommended a synchronized switch from trivalent oral polio vaccine (tOPV) to bivalent oral polio vaccine (bOPV) and inactivated polio vaccine (IPV) use in routine introduction, but added that this would require very accurate, detailed, timely, and global project planning, with regional as well as global consensus. In 2012, the World Health Assembly declared the completion of the eradication of poliomyelitis as a global public health programmatic emergency, and a Polio Eradication and Endgame Strategic Plan was developed. The second objective of the plan is the phased removal of oral polio vaccines (OPVs), starting with vaccine containing poliovirus type 2 (OPV2) and introduction of at least 1 dose of IPV in routine immunization as a risk mitigation measure. Use of IPV is intended to boost systemic immunity and to eliminate the rare risks of vaccine-associated paralytic polio and circulating vaccine-derived poliovirus (cVDPV).

An additional objective of the endgame strategy is to strengthen routine immunization (RI) as the primary pillar to sustain high population immunity. Routine immunization performance will be strengthened through implementation of activities and strategies to raise immunization coverage and to ensure high population immunity against polioviruses, especially type 2, after withdrawal of OPV2 [2]. Outbreaks of wild poliovirus (WPV) and cVDPV are closely correlated with low vaccine coverage and hence routine immunization performance [3]. An opportunity was therefore presented to use the Global Polio Eradication Initiative (GPEI) infrastructure to further strengthen RI and improve immunization coverage to sustain a polio-free world.

Over the years progress was made in the African region and transmission of WPV was interrupted and, for >24 months, no case of WPV was reported [4, 5] until the recent cases in Nigeria. Nigeria was therefore delisted as an endemic country in September 2015 [6–10]. Specifically, WPV type 2 (WPV2) has not been reported since 1999, and WPV type 3 was last detected 11 November 2012 [10, 11].

This significant progress was made possible through widespread use of mainly tOPV, consisting of types 1, 2, and 3 live, attenuated polioviruses [11–13]. The use of tOPV is despite the chances of the vaccine viruses undergoing genetic changes during intestinal replication and resulting in vaccine-derived polioviruses (VDPVs) capable of causing paralytic polio in communities with low vaccination coverage [14]. Elimination of the risk of VDPVs requires global stoppage of OPV use as an imperative. Confirming the risk posed by the use of OPV, Hampton noted that among 686 cases of paralytic polio caused by cVDPVs, detected since 2006, type 2 cVDPVs (cVDPV2) accounted for >97% [11]. Thus, elimination of the risks posed by cVDPV2 demands the withdrawal of OPV2 through a synchronized replacement of all tOPV with bOPV containing only types 1 and 3 polioviruses [15, 16]. This switch from tOPV to bOPV was therefore scheduled for April 2016 [16].

As of 24 June 2015, 90 (46%) of 194 WHO member states were using IPV, 102 (53%) had established dates for the introduction of IPV, and 2 (1%) intended to introduce IPV in 2015 but had not set dates for doing so. In the WHO African Region, all 47 member states were using tOPV in RI, but a few countries had used bOPV and Kenya used IPV in campaigns.

In April 2015, the Strategic Advisory Group of Experts on immunization (SAGE) in its recommendation set April 2016 as the date for the switch, stressing that any delays will only be considered on the grounds that the risk for continued cVDPV2 transmission is deemed to be high by October 2015 [16]. Therefore all 47 countries of the WHO African Region switched from tOPV to bOPV. This report summarizes the introduction of IPV and preparations for the switch from tOPV to bOPV, highlighting the lessons learned and the challenges overcome.

## STRENGTHENING ROUTINE IMMUNIZATION AS ESSENTIAL PART OF SWITCH TO BOPV

Routine immunization is a major pillar of the GPEI endgame strategic plan, delivering 4 doses of tOPV at birth, 6, 10, and 14 weeks of age. To sustain the gains of polio eradication and maintain a polio-free African Region, resources of the polio eradication initiative were deployed to improve RI performance and to increase immunization coverage in several countries [2, 17]. Financial, physical, human, and other resources were used to support immunization programs. Systems were put in place to monitor the progress of national plans through teleconferences and face to face meetings.

Microplanning was used to identify settlements or villages with a high number of unvaccinated children and a global positioning systems (GPS) was used to create accurate, coordinate-based maps for polio-endemic states that were consequently utilized to provide routine vaccines [17]. For instance, health facility microplans were updated in what is named in Nigeria “reach every ward” to capture previously unreached settlements, improving access and utilization of the immunization services by reestablishing immunization service delivery points (fixed sites) in wards with existing facilities and outreach or mobile sites [2]. Strategies used for unreached children during polio campaigns were leveraged to ensure that RI services are provided in identified areas. In addition to delivering OPV, the national or subnational immunization days and child days were used as opportunities to administer other routine vaccines in all countries in addition to the micronutrient vitamin A, iron, and insecticide-treated bednets.

Furthermore, some of the staff whose positions were funded by the polio eradication program provided training to health workers in immunization. Data were analyzed and used for actions to improve performance at all levels. Similarly, GPEI contributions to supportive supervision was strengthened and text messaging was used to transmit data and drive corrective actions, in what is known as Nigeria’s real-time tracking of routine immunization supervision [2]. Regular meetings were also held between the village committees and health facility staff to improve linkages and enable good turnout during outreach and mobile services. Community volunteers were trained to support health workers in revising microplans to identify new outreach points.

Program management, processes, human and financial resources, vehicles, and tools were also used in managing the routine Expanded Programme on Immunization (EPI). GPEI led to monthly monitoring and decision-making meetings that took into account immunization and existing problems in the health sector. In Tanzania, health workers with experience in polio management provided RI and other services in an integrated manner in remote villages that lacked adequate health services [2]. GPEI funds facilitated the logistics and management of vaccines ensuring uninterrupted supply of vaccines at the endpoints. With examples from Ethiopia and Chad, Table 1 further illustrates how polio assets, systems, and infrastructures were deployed to strengthen routine immunization in the African Region. The situation in Chad and Ethiopia was typical of all the countries, with GPEI investments in the region with respect to strengthening RI systems.

**Table 1.** Use of Polio Assets in Strengthening Routine Immunization in the African Region: Examples From Ethiopia and Chad

Domains of GPEI Expertise	Ethiopia	Chad
1. Policy and strategy development	• cMYP 2016–2020 to be elaborated integrating RI activities, GPEI, new vaccines introduction • Routine EPI improvement plan with a focus on high-risk zones • IPV incorporated in revised EPI policy guidelines	• Development of the cMYP 2015–2017 integrating RI and NV introduction, GPEI, and other accelerated disease initiatives and surveillance of VPD
2. Planning	• 2015 annual work plan with 6-month roadmap • Integrated microplanning with polio, nonpolio SIAs, and routine service delivery. • Polio surge capacity support planning for routine delivery services and nonpolio campaigns	• 2015 work plan with 6-month roadmap • Integrated microplanning using appropriate template
3. Management and oversight	• ICC and polio command posts at central level provide oversight and orientation on immunization activities and monitor implementation • Establishment of NITAG • Quarterly EPI review meeting with all regions at national level (integrated RI and polio discussions)	• ICC and polio TAG at national level • Monthly meetings with the head of state • Biannual monitoring meeting in the 6 hubs • Monthly monitoring meeting in the 54 RED districts
4. Implementation and service delivery	• High-risk zones for focused support identified • Specific strategy to reach pastoralists and hard-to-reach areas • Best practices development and documentation • Use of GPEI network for the best community mapping during microplanning • Provision of integrated package of vaccines in remote and hard-to-reach areas	• High-risk zones and population (nomads, security compromised areas) included in the microplan • Best practices documentation • Use of GPEI network for the best community mapping during microplanning
5. Monitoring and evaluation	• Weekly conference calls with IST/AFRO and Somali region to monitor the level of implementation of activities with partners • Monthly RI report on immunization including surveillance • Feedback at lower level for corrective actions • Quarterly supervision missions to regions and priority zones	• Weekly conference calls with IST/AFRO to monitor the level of implementation of activities with partners • SITREP polio including RI and other accelerated disease initiatives • Monthly feedback bulletin shared with lower level • Dashboard to monitor key immunization indicators
6. Communications and community engagement	• Integrated communication strategy exists • Keys messages dissemination through multiple channels • Printing of IEC materials including jobs aids for health workers in different languages	• Communication plan integrated in the 2015 EPI plan and including demand creation, community engagement, advocacy, and strategies for high risk and underserved
7. Surveillance and data analysis	• Integrated VPD surveillance activities conducted by polio funded field staff • Weekly update on VPD surveillance indicators and feedback to lower level and partners • Quarterly monitoring dashboard of indicators and officers’ performance • Outbreak response activities	• Weekly/monthly monitoring of surveillance indicators • Monthly analysis of data to orient decision (SITREP), monthly feedback bulletin on immunization • Planning and implementation of outbreak response
8. Capacity building	• Development/ updating of training materials • Training activities in focus zones and regions (MLM, IIP, disease surveillance) • Human resources involved in EPI/other immunization activities is same as the one involved in polio eradication	• Development of training material • Training activities conducted at all level supported by the hubs (IIP, MLM, surveillance, data quality, etc)
9. Partnerships and coordination	• ICC and command post at national level holds biannual/ quarterly meetings • Quarterly review meeting with all regions at national level	• Weekly meetings of the technical ICC

Abbreviations: cMYP, comprehensive multiyear plan; EPI, Expanded Programme on Immunization; GPEI, Global Polio Eradication Initiative; ICC, intercountry coordinating committee; IEC, Information Education and Communication; IIP, Immunization in Practice; IPV, inactivated polio vaccine; IST/AFRO, Intercountry Support Team of the African Regional Office; MLM, mid level manpower; NITAG, National Technical Advisory Group; NV, new vaccine; RED, reach every district; RI, routine immunization; SIAs, supplemental immunization activities; SITREP, situation report; TAG, Technical Advisory Group; VPD, vaccine-preventable diseases.

**Figure 1.**
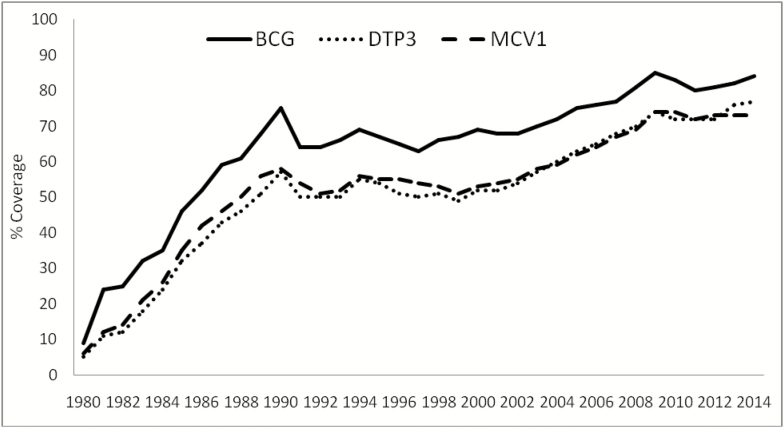
Trends in coverage of some vaccines in the African Region. Abbreviations: BCG, bacillus Calmette-Guérin; DTP3, third dose of diphtheria, tetanus and pertussis containing vaccine; MCV1, first dose of measles containing vaccine.

These efforts resulted in improved performance of RI in the region. As a result, national infant immunization coverage rates for bacillus Calmette-Guérin (BCG), third dose of vaccine containing diphtheria, tetanus and pertussis (DTP3), and first dose of measles containing vaccine (MCV1) rose steadily between 2011 and 2014 (Figure 1) in the region. Specifically in some of the countries with huge polio investments such as the Democratic Republic of Congo (DRC) and Nigeria, DTP3 coverage rose to 80% and 66% from <50% and <40%, respectively, in the period under review. In Angola, Chad, and Togo, DTP3 coverage reached 87%, 46%, and 87%, respectively. A moderate increase was also observed in Cote d’Ivoire (58% to 67%), Tanzania (82% to 97%), and Ethiopia (55% to 77%). The regional coverage for DTP3 vaccine increased from 5% in 1980 to 77% in 2014.

Anya et al further demonstrated that of the 47 countries in the region, 18 countries (38%) achieved a national coverage for DTP3-containing vaccine of ≥90% meeting the global and regional immunization targets in 2014 (Table 2) [2]. An increase in national vaccination coverage was reported in Ethiopia, DRC, and Nigeria, whereas a decrease was noted in 17 countries, including the 3 Ebola-affected countries (Guinea, Liberia, and Sierra Leone).

**Table 2.** World Health Organization (WHO)/United Nations Children’s Fund Estimate of Vaccination Coverage for Selected Expanded Programme on Immunization Antigens by Country, WHO African Regional Office, 2014

Countries	DTP3	MCV1	OPV3	IPV Introduction Into Routine	
				Status	Dated
Algeria	95	95	95	Introduced	December 2015 Q4, 2015
Angola	80	85	81	Planned	Q4 2017
Benin	70	63	72	Introduced	August 2015
Botswana	95	97	96	Introduced	November 2015
Burkina Faso	91	88	91	Planned	Q4 2017
Burundi	95	94	95	Introduced	November 2015
Cabo Verde	95	93	95	Planned	Q4 2017
Cameroon	87	80	86	Introduced	July 2015
CAR	47	49	47	Introduced	September 2015
Chad	46	54	54	Introduced	August 2015
Comoros	80	80	79	Introduced	January 2015
Congo	90	80	90	Introduced	April 2016
Côte d’Ivoire	67	63	66	Introduced	June 2015
DRC	80	77	79	Introduced	April 2015
Equatorial Guinea	24	44	30	Introduced	August 2016
Eritrea	94	96	94	Planned	Q4 2017
Ethiopia	77	70	75	Introduced	December 2015
Gabon	70	61	68	Introduced	December 2015
The Gambia	96	96	97	Introduced	April 2015
Ghana	98	92	98	Planned	Q4 2017
Guinea	51	52	42	Introduced	November 2015
Guinea-Bissau	80	69	78	Introduced	July 2016
Kenya	81	79	81	Introduced	December 2015
Lesotho	96	92	95	Introduced	April 2016
Liberia	50	58	49	Planned	Q4 2017
Madagascar	73	64	73	Introduced	May 2015
Malawi	91	85	87	Planned	Q4 2017
Mali	77	80	84	Introduced	March 2016
Mauritania	84	84	84	Introduced	November 2015
Mauritius	97	98	98	Introduced	November 2015
Mozambique	78	85	78	Introduced	November 2015
Namibia	88	83	88	Introduced	November 2015
Niger	68	72	67	Introduced	July 2015
Nigeria	66	51	66	Introduced	February 2015
Rwanda	99	98	99	Planned	Q4 2017
Sao Tome & Principe	95	92	95	Introduced	April 2016
Senegal	89	80	85	Introduced	January 2015
Seychelles	99	99	99	Introduced	September 2015
Sierra Leone	83	78	83	Planned	Q4 2017
South Africa^a^	70	70	71		
South Sudan	39	22	44	Introduced	December 2015
Swaziland	98	86	98	Introduced	July 2016
Togo	87	82	85	Planned	Q4 2017
Uganda	78	82	82	Introduced	April 2016
Tanzania	97	99	97	Planned	Q4 2017
Zambia	86	85	78	Planned	Q4 2017
Zimbabwe	91	92	92	Planned	Q4 2017

Abbreviations: CAR, Central African Republic; DRC, Democratic Republic of Congo; DTP3, third dose of diphtheria, tetanus and pertussis containing vaccine; IPV, inactivated polio vaccine; MCV1, first dose of measles containing vaccine; OPV3, third dose of oral polio vaccine; Q4, quarter 4.

^a^South Africa has been using hexavalent vaccine, which includes IPV. South Africa is the only country in the region that introduced IPV before the Endgame strategy

**Figure 2.**
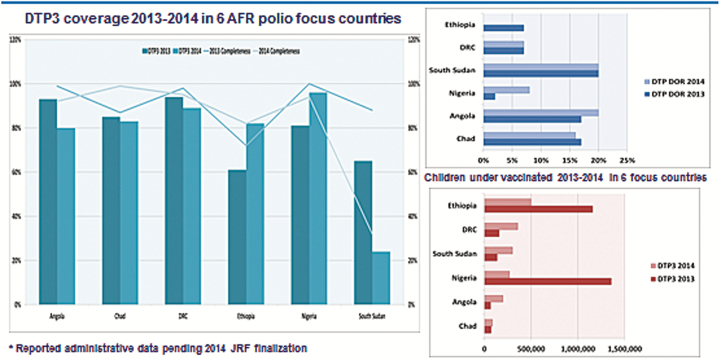
DTP dropout rates in 6 focused countries in the African Region, 2013–2014. Abbreviations: AFR, African Region; DOR, dropout rate; DRC, Democratic Republic of Congo; DTP3, third dose of diphtheria, tetanus and pertussis containing vaccine; JRF, WHO UNICEF joint reporting format.

**Figure 3.**
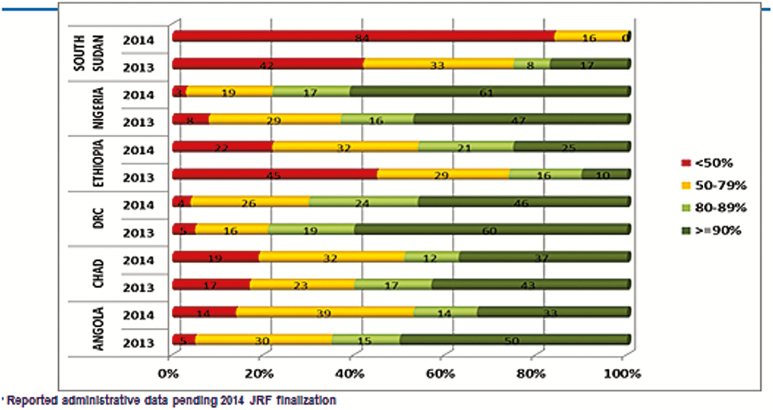
DTP3 district performance in 6 focus countries, 2013–2014. Abbreviations: DRC, Democratic Republic of Congo; JRF, WHO UNICEF joint reporting format.

A further analysis of results from 6 countries in Africa, following the strengthening of RI using GPEI systems and infrastructures, revealed significant improvements in the RI programs in Angola, Chad, DRC, Ethiopia, Nigeria, and South Sudan in 2014. Figure 2, for instance, showed a significant reduction in dropout rates in the 6 countries. Similarly, Figure 3 revealed some improvements on DTP3 coverage at the district level in the 6 focus countries. For instance, fewer districts recorded <50% coverage in Nigeria, Ethiopia, and DRC in 2014 compared with 2013. On the other hand, more districts in Nigeria and Ethiopia recorded ≥90% coverage in 2014 compared to 2013 [2].

## REGISTRATION OF VACCINES AND IMPORT LICENSES PRIOR TO INTRODUCTION OF IPV

The standard pathway for vaccine registration requires the submission of a Common Technical Document to a regulatory authority of origin, who will review it and provide approval for a marketing authorization before the vaccine can be introduced or exported to other countries [18]. The national regulatory authority of the country of importation is also required to register the vaccine and to issue a permit to the manufacturer for vaccine shipment. The countries of the African region have regulatory authorities, legally mandated to fulfill all the regulatory functions including the registration of all health products, including vaccines before they are shipped into the countries to be introduced. One of the challenges facing timely introduction of new vaccines is the weak capacity of countries of the WHO African Region to register vaccines and to issue an import permit prior to shipment of vaccine for use in countries.

Most vaccines, including IPV and OPV, were developed in countries outside the region, fully licensed by national regulatory authorities of these countries and prequalified by WHO. Most countries in the African Region take into account the work carried by the WHO prequalification in reviewing or providing regulatory approval for the importation and use of new vaccines. The WHO expedited review procedure [2] and the collaborative procedure [19] represent fast-track methods recommended by who for the registration of prequalified vaccines.

To address the challenges and ensure that both IPV and bOPV are duly licensed and available in all the countries as an urgency to meet the switch timelines, countries were supported by WHO in identifying a suitable pathway for the registration of IPV and bOPV. The technical support that was provided ensured that there was no delay in shipment of the right quantities of vaccines and the subsequent synchronized switch. WHO gathered information from each of the 47 countries of the African Region on how vaccines have been registered previously or whether a waiver was given for importation and use. This included 16 countries (Angola, Burundi, Cape Verde, Comoros, Equatorial Guinea, Eritrea, Gambia, Guinea-Bissau, Lesotho, Liberia, Malawi, Namibia, Sao Tomé e Principe, Seychelles, Sierra Leone, Swaziland) that, in principle, accepted WHO-prequalified bOPV, without resorting to a dossier submission by the manufacturers and a review, either through an expedited pathway or a regular full review. Angola, had already given approvals or waivers for the importation and use of bOPV in supplemental immunization activities. The data showed that several countries which responded to WPV outbreaks had in the past used bOPV and Kenya used IPV in campaigns. The information that was obtained formed the basis for guidance and support to enable the countries register these vaccines ahead of the switch.

All the countries provided permits for the importation of IPV and bOPV, in time for the use of IPV and the switch to bOPV. Follow-up was instituted by WHO and the United Nations Children’s Fund (UNICEF) on a regular basis to ensure that the vaccines were registered and the means by which the decision was made was also identified and documented. Two joint reviews using the WHO collaborative procedure were organized for groups of Francophone and Anglophone countries, to facilitate licensure of IPV and bOPV.

## INTRODUCTION OF IPV INTO ROUTINE IMMUNIZATION PROGRAMS

Training and sensitization is important as part of any new vaccine introductions, even for health workers with experience in immunization. Therefore, the WHO Regional Office for Africa and UNICEF co-organized 2 workshops in April 2014 for participants drawn from the English-speaking (3–4 April 2014) and French-speaking (14–15 April 2014) countries in the African Region. Each 2-day workshop equipped participants with up-to-date technical information and references to guide the interaction with decision makers and program managers, and to train a critical mass of consultants to support country planning activities and training sessions for the introduction of IPV.

Specifically, participants received knowledge about poliomyelitis, the eradication efforts, existing and expected vaccines, and the endgame strategy. They also learned about logistics, operational, regulatory, and communication issues. Furthermore, the workshops emphasized the role of routine immunization as one of the tripods in the eradication of polio in the region. Participants went through routine immunization systems strengthening and how to leverage the polio infrastructure and resources to strengthening routine immunization for a sustainable fight against polio and other vaccine-preventable diseases. Participants also discussed the policies and operational procedures of Gavi, the Vaccine Alliance, as well as the process of applying to Gavi funding for the introduction of IPV into RI.

The trained personnel were deployed to support the countries as they prepared for the introduction of IPV into RI. Thirty-three countries (Table 2) successfully introduced IPV into RI, ready to switch from tOPV to bOPV. However, because of the global IPV supply constraint, the remaining 13 countries will have to wait until the end of 2016 to introduce the vaccine.

## SWITCH FROM TOPV TO BOPV IN THE AFRICAN REGION

In compliance with the World Health Assembly mandate, a strict window of between 15 April and 1 May 2016 was set for the African Region to stop the use of OPV beginning with removal of the type 2 component of tOPV through a coordinated switch to bOPV, containing only types 1 and 3 As a first step toward meeting this target, all OPV-using countries in the African Region started planning in earnest during the second quarter of 2015 and finalized budgeted National Switch Plans by the last quarter of 2015.

A series of national level “dry runs” was undertaken to test global guidance materials; to learn from a desk-based walkthrough of the steps to carry out the switch and explore areas of weakness, gaps, barriers; and to identify potential facilitators and solutions. The switch dry runs helped in informing the finalization of the global guidance to countries on planning for and implementing the switch. Countries benefited from these advanced preparation of their switch plans, including identification of the critical roles and responsibilities of national regulatory authorities, EPI, ministry of health, and partner organizations in each of the planning, preparation, implementation, and validation phases of the switch.

In the African Region, UNICEF, Centers for Disease Control and Prevention, Clinton Health Access Initiative (CHAI), Bill & Melinda Gates Foundation, Gavi, and WHO provided technical and other support to the countries in planning, preparation, implementation, and validation of the switch. The 2 organizations collaboratively organized workshops to build the capacities of the countries to conduct the switch. Participants were given orientation on global update on the strategic plan for the eradication of polio and the final phase; guidance on operational planning of the implementation, monitoring, and validation; tools and various materials for training, logistics, monitoring and validation; and the process of monitoring and validation of the switch. Country preparedness was assessed with a questionnaire containing 12 questions, which was administered on the first day of the workshop.

The implementation was done in 4 phases: planning, preparation, implementation, and validation [20, 21]. The planning commenced with the creation of national regional and district switch management committees. The National Switch Committee had the responsibility for selecting a national switch day; developing a national plan and establishing subcommittees for logistics, communication, and process monitoring; identifying focal points; and establishing an operations center. There was a National Switch Validation Committee responsible for validating the withdrawal of the tOPV from national, regional, and district vaccine stores, and from service delivery points. The tOPV inventory and procurement plan as well as planning for bOPV acquisition, including obtaining regulatory approval and import permit, and the distribution were conducted at this level.

There were also mechanisms such as securing funds for the switch and developing and implementing communications strategy. The management of logistics included the assessment of cold chain capacity, determining disposal strategy, and developing training materials for logisticians and health workers as well as the establishment of monitoring system. Switch management committees at national and regional levels selected, monitored, and reported on indicators and achievements [20, 21]. Table 3 highlights the outcomes of the monitoring exercises in countries.

**Table 3.** Independent Monitoring of Switch Outcome in the African Region

Country	HFs, No.	% of HFs Monitored^a^	% of HFs With tOPV in Cold Chain	% of HFs With bOPV	% of HFs With IPV	Total tOPV Vials Collected for Disposal^b^	Validation Dates
Benin	95	100.0	7.0	97.0	91.0	NC	10 May 2016
Botswana	674	29.4	0.0	99.5	93.9	14682	26 April 2016
Burkina Faso	1920	12.0	0.0	100.0	NA	21502	17 May 2016
Cabo Verde	38	100.0	0.0	87.0	0.0	4147	4 May 2016
Cameroon	992	103.5	2.4	80.8	78.5	266528	12 May 2016
CAR	316	48.0	3.0	66.0	71.0	NC	11 May 2016
Chad	1341	23.8	3.2	91.5	94.0	479300	16 May 2016
Comoros	5	100.0	0.0	100.0	100.0	NC	6 May 2016
Republic of Congo	251	16.0	0.0	82.0	82.0	37094	9 may 2016
Cote d’Ivoire	2007	19.0	0.0	88.0	93.0	20339	28 April 2016
DRC	6416	28.0	0.0	35.0	43.0	87908	14 May 2016
Equatorial Guinea	47	100.0	4.0	98.0	NA	NC	13 May 2016
Eritrea	295	25.0	0.0	100.0	NA	22860	20 May 2016
Ethiopia	3990	50.4	0.3	78.0	76.6	77252	19 May 2016
Gabon	59	100.0	0.0	100.0	95.0	NC	9 May 2016
The Gambia	69	28.0	0.0	100.0	100.0	NC	4 May 2016
Ghana	3649	11.9	1.2	96.8	NA	31084	9 May 2016
Guinea	439	47.0	10.0	87.0	87.0	NC	14 May 2016
Guinea-Bissau	116	52.0	0.0	100.0	NA	9638	5 May 2016
Kenya	6121	18.3	6.0	92.6	90.2	366264	30 April 2016
Lesotho	200	100.0	24.0	93.0	93.0	8983	11 May 2016
Liberia	534	53.0	4.0	97.5	NA	376360	2 May 2016
Madagascar	2598	19.0	1.0	68.0	67.0	386918	12 May 2016
Malawi	823	10.0	0.0	100.0	NA	12848	10 May 2016
Mali	1423	28.0	5.0	83.0	86.0	137000	11 May 2016
Mauritania	NA	100.0	NA	NA	NA	50744	17 May 2016
Mauritius	29	100.0	0.0	100.0	100.0	383	16 May 2016
Mozambique	1461	38.0	0.0	100.0	80.0	265027	19 May 2016
Namibia	443	38.0	1.1	100.0	100.0	6187	10 May 2016
Niger	978	12.0	1.7	100.0	100.0	1843053	17 May 2016
Nigeria	25651	12.0	2.0	93.0	50.0	3553693	29 April 2016
Rwanda	507	20.0	0.0	100.0	NA	11670	13 May 2016
Sao Tome and Principe	21	100.0	5.0	100.0	95.0	33600	4 May 2016
Senegal	1041	16.0	1.2	95.0	99.0	NC	17 May 2016
Seychelles	14	100.0	0.0	100.0	100.0	7120	18 May 2016
Sierra Leone	785	11.0	4.0	94.0	NA	78477	3 May 2016
South Africa	4824	29.0	3.9	92.0	76.0	168150	3 May 2016
South Sudan	399	67.0	7.0	62.0	72.0	37882	20 May 2016
Swaziland	177	100.0	0.0	100.0	NA	NC	9 May 2016
Tanzania	5999	20.4	0.2	98.5	NA	1516	3 May 2016
Togo	712	29.0	0.0	100.0	NA	NC	29 April 2016
Uganda	2747	74.9	7.2	92.8	91.8	41012	13 May 2016
Zambia	1904	12.0	0.0	100.0	NA	28560	13 May 2016
Zimbabwe	1647	10.3	0.6	99.4	NA	4295	10 May 2016

Abbreviations: bOPV, bivalent oral polio vaccine; CAR, Central African Republic; DRC, Democratic Republic of Congo; HF, health facility; IPV, inactivated polio vaccine; NA, not available; NC, not compiled; tOPV, trivalent oral polio vaccine.

^a^In addition to health facilities, all vaccine stores were monitored. There was no tOPV found in any of the primary vaccine stores, but some were found in the district store in 6 of 20 countries.

^b^Botswana, Eritrea, Ethiopia, Malawi, Namibia, Rwanda, Seychelles, South Africa, South Sudan, Swaziland, Uganda, Tanzania, Zambia, and Zimbabwe have submitted official report on the disposal and all used high-temperature incineration.

Implementation of the switch started with training of switch monitors on their roles and responsibilities as well as the modus operandi of the switch. They were also trained on methods for confirming the absence of tOPV at vaccine stores and selected service delivery points, removing tOPV if any is found in facilities and reporting to supervisors. On the other hand, the training of health workers on the important aspects of the switch such as the rationale for the switch as well as setting of switch date, and monitoring were systematically undertaken.

Communication and media messages followed the standard switch protocol. The messages covered such information as “All tOPV should be removed from the cold chain, packed in bags with sticker on which it is written “Do not use/For disposal.” The remaining tOPV should be destroyed or securely stored within 3 months following the national switch date. The validation was carried out by existing Polio Eradication Certification Committees and covered a 2-week period postswitch. Based on data collected by switch independent monitors, the National Switch Validation Committee validated tOPV withdrawal, and submission of the ministry of health endorsed the switch validation report submitted to WHO by the countries.

The process revealed that governments provided funds for the switch at the operational levels. The tOPV collection bags and stickers were also available. However, microplanning of independent monitors and selection of independent monitors and supervisors had a low level of achievement. Switch committees exited at the different levels of operation. The GPEI also allocated funds to the switch. The second order of bOPV and planning of training for independent monitors had a proportion of achievement from 50% to 79%. The National Switch Validation Committee was in place in all the countries and conducted the identification of tOPV destruction sites, and the training of logisticians and health workers had a very high level of attainment.

The West African situation was replicated in both the East and Southern African and the Central African subregions. Various committees and resources were in place, and the identification of destruction sites and logistics were carried out satisfactorily. However, unlike the situation in West Africa, availability of the financial resources allocated by the GPEI and selection of independent monitors and supervisors had a medium level of achievement. Availability of national Switch validation committee and planning of independent monitors training had a high level of attainment.

## DISCUSSION

The global switch from tOPV to bOPV represented a significant milestone in the effort to eradicate polio in the African Region, because it marked the eradication of WPV2 and, in the long term, should lead to the elimination of type 2 VDPVs [11]. Careful synchronization of the switch from tOPV to bOPV within and across OPV-using countries was observed with all 47 member states conducting the switch as recommended.

The entire exercise was a success for a number of key reasons, and some valuable lessons were learned. The anticipation and establishment of a committee of experts in 2006 to examine closely the pre– and post–polio eradication issues and to advise the organization was important, as it contributed to the discussions by SAGE and the final recommendation on the switch. This further underscores the vital role of early planning and working across the entire organization—headquarters to region, to intercountry support teams, and to countries where most of the work was carried out.

The switch could not have succeeded without the close collaboration and partnership between UNICEF and WHO at the subnational, national, regional, and global levels. Each organization brought its expertise and strengths to bear on the task of supporting the countries and there was fine coordination leading to the success. Face-to-face meetings were supplemented by regular teleconference calls and frequent exchange of emails. It was important for technical and other support to be well coordinated between the 2 organizations, for activities to be planned together, and for any potential issues to be managed. The committee that examined the pre– and post–polio eradication issues in the WHO African Region was derived from the membership of the Task Force on Immunization, now called the Regional Immunization Technical Advisory Group, which is set up and mandated to advise the Regional Director on Vaccines and Immunization. It did not include any external organization.

Adequate preparations often produce a better chance of success in public health action. Following the World Health Assembly resolution, a clear strategy for the endgame, SAGE recommendations, and regional and national plans were put in place. It was crucial for each country to decide on a date for the switch, within the SAGE-recommended period, communicate this to all stakeholders, and make preparations to implement the switch.

Training was also effective, regardless of how knowledgeable health workers in a country were. The training ensured that status of preparations could be monitored and remedial action taken where delays in implementation were imminent. The role of training can therefore not be underestimated in such a major change in immunization since the introduction of the EPI.

By bringing countries together, experiences and best practices were shared, knowledge passed on, and mentorships established; all countries were inspired to aim at achieving a smooth switch. The outcome, unsurprisingly, was that all countries met the recommendations and provided validation of the switch on time.

The success was also due to having reliable data, which was analyzed and used for decision making. The data on routine immunization performance dictated the level of technical support required for each country in the region, bearing in mind that immunization systems vary in strength, just as health systems in general do across the 47 countries of the WHO African region. The data were used for national planning, acceptability of prequalification vaccines, national pathways for registration of vaccines, informing on tOPV levels, cold chain capacity, target populations, and other key considerations.

However, it is well recognized that cVDPV2 outbreaks, caused either by circulating strains and/or newly emerged strains, could occur after the switch given that a high proportion of persons susceptible to infections with type 2 polioviruses will increase over time from new birth cohorts not receiving tOPV and because multiple low-income countries already have low polio vaccination coverage [22]. As a result, following the switch from tOPV to bOPV, reducing the likelihood and potential extent of cVDPV2 outbreaks is essential, as is the ability to detect and respond to any such outbreaks that do occur [11].

Furthermore, for the reason of averting an explosion in the outbreak of cVDPVs, countries planned and introduced IPV in their RI systems to boost herd immunity. The introduction of IPV should aid in preventing paralytic polio from wild or vaccine-derived type 2 polioviruses in many persons who have received only bOPV by providing them immunity to type 2 viruses [11]. Thirty-three countries in the African Region introduced IPV into their RI systems.

To support the introduction of IPV as well as sustain the gains of polio eradication, polio resources were deployed to strengthen the RI systems. The health workers were trained and logistics supplies enhanced. Polio systems and accountability structures as well as planning techniques were deployed in managing the RI system. The RI structure ensured smooth distribution and administration of the IPV and, in situations of limited supplies, prioritized IPV for areas at high risk for cVDPV2 outbreaks to enhance the impact of IPV use. The GPEI supported the IPV introduction, including technical assistance and funding for IPV purchases and operational expenses in all countries.

However, the use of IPV alone is not capable of effectively preventing the spread of poliovirus infections, as was the case in recent repeated isolation of type 1 WPVs through environmental surveillance in Israel, where the population had high IPV coverage, but, because OPV had not been used since 2004, silent circulation of introduced wild polioviruses occurred [11, 23]. As tOPV is withdrawn, efforts are made in the region to ensure high-quality surveillance for circulating polioviruses, both through acute flaccid paralysis surveillance and environmental surveillance, and structures are in place to promote prompt, aggressive responses to any identified type 2 poliovirus outbreaks.

In conclusion, the switch passed off smoothly due to early planning; coordination of support by UNICEF, WHO, and partners; and national efforts. To address the challenges, efforts should be made to further strengthen routine immunization and improve the quality of acute flaccid paralysis surveillance, environmental surveillance, and the preparedness to respond robustly and effectively to any outbreaks of polio.

## Notes

***Supplement sponsorship.*** This work is part of a supplement coordinated by the Task Force for Global Health with funding provided by The Bill and Melinda Gates Foundation and the Centers for Disease Control and Prevention.

***Potential conflicts of interest.*** All authors: No reported conflicts of interest. All authors have submitted the ICMJE Form for Disclosure of Potential Conflicts of Interest. Conflicts that the editors consider relevant to the content of the manuscript have been disclosed.
